# Genetic and Phenotypic Heterogeneity in Chinese Patients with Waardenburg Syndrome Type II

**DOI:** 10.1371/journal.pone.0077149

**Published:** 2013-10-23

**Authors:** Shuzhi Yang, Pu Dai, Xin Liu, Dongyang Kang, Xin Zhang, Weiyan Yang, Chengyong Zhou, Shiming Yang, Huijun Yuan

**Affiliations:** 1 Department of Otolaryngology, The First Affiliated Hospital of Chinese PLA General Hospital, Beijing, China; 2 Institute of Otolaryngology, Chinese PLA General Hospital, Beijing, China; 3 Department of Otolaryngology, The 16^th^ Hospital of Chinese PLA, Alatai, Xinjiang Autonomous Region, China; Charité Universitätsmedizin Berlin, NeuroCure Clinical Research Center, Germany

## Abstract

Waardenburg Syndrome (WS) is an autosomal-dominant disorder characterized by sensorineural hearing loss and pigmentary abnormalities of the eyes, hair, and skin. Microphthalmia-associated transcription factor (*MITF*) gene mutations account for about 15% of WS type II (WS2) cases. To date, fewer than 40 different *MITF* gene mutations have been identified in human WS2 patients, and few of these were of Chinese descent. In this study, we report clinical findings and mutation identification in the *MITF* gene of 20 Chinese WS2 patients from 14 families. A high level of clinical variability was identified. Sensorineural hearing loss (17/20, 85.0%) and heterochromia iridum (20/20, 100.0%) were the most commonly observed clinical features in Chinese WS2 patients. Five affected individuals (5/20, 25.0%) had numerous brown freckles on the face, trunk, and limb extremities. Mutation screening of the *MITF* gene identified five mutations: c.20A>G, c.332C>T, c.647_649delGAA, c.649A>G, and c.763C>T. The total mutational frequency of the *MITF* gene was 21.4% (3/14), which is significantly higher than the 15.0% observed in the fair-skinned WS2 population. Our results indicate that *MITF* mutations are relatively common among Chinese WS2 patients.

## Introduction

Waardenburg syndrome (WS) is an autosomal-dominant disorder of neural crest cell differentiation, most commonly described in Western populations. Its main clinical manifestations include sensorineural hearing loss, pigmentary abnormalities of the eyes, hair, and skin (*e*.*g*., heterochromia iridum, white forelock, and patchy hypopigmented skin), and dystopia canthorum [1], [2]. Four types of WS have been identified, depending on their clinical characteristics. WS type I (WS1; MIM 193500) and type II (WS2; MIM 193510) are distinguished by the presence or absence, respectively, of dystopia canthorum. The presence of limb abnormalities distinguishes type III (WS3; also called Klein-Waardenburg syndrome; MIM 148820) from WS1. Type IV (WS4; also called Shah-Waardenburg syndrome or Waardenburg- Hirschsprung disease; MIM 277580) is characterized by the presence of an aganglionic megacolon. Of these subtypes, WS1 and WS2 are the most common.

WS2 is well-defined phenotypically and is genetically heterogeneous; its hallmarks are sensorineural hearing loss and heterochromia iridum. Other abnormal pigmentation disturbances, including white forelock, early graying, and hypo- or hyperpigmented skin patches, are also manifested in relatively low proportions of WS2 patients [3]. Five subtypes of WS2 have been identified based on molecular findings. WS2A (MIM 193510) is caused by *MITF* mutations [4]. Mutations in *SNAI2* (snail homolog of 2) gene are known to cause WS2D (MIM 608890) [5], and mutations in *SOX10* (SRY (sex-determining region Y)-box10) gene are responsible for WS2E (MIM 611584) [6]. WS2B (MIM 600193) maps to chromosome 1p [7] and WS2C (MIM 606662) maps to chromosome 8p [8], but the causative genes for these have not yet been identified. To date, about 30% of WS2 cases can be explained at the molecular level. Some researchers have proposed that the *MITF* gene is responsible for approximately 15% of WS2 cases and the *SOX10* gene for approximately another 15% [4], [6]. A homozygous deletion in the *SNAI2* gene was described in two unrelated WS2 patients [5], and a heterozygous endothelin receptor type-B (*EDNRB*) mutation has been found in three patients from one family [9]. However, no other patients have been identified to confirm these results, indicating that *SNAI2* and *EDNRB* are not major causes of WS2 [4]–[9]. In this study, we conducted detailed analyses of the clinical manifestations and molecular bases of 20 Chinese WS2 patients from 14 families. Specifically, we examined three WS2-related genes: *MITF*, *SOX10*, and *SNAI2*.

## Materials and Methods

### Patients and DNA samples

The subjects for this study were recruited from the Otology Clinic at Chinese PLA General Hospital and deaf-mute schools from nine different regions in China. The study was approved by the ethics committee of the Chinese PLA General Hospital. Written informed consent was obtained from all adult subjects and guardians on behalf of the children prior to the clinical evaluation and blood sample collection.

**Figure 1:**
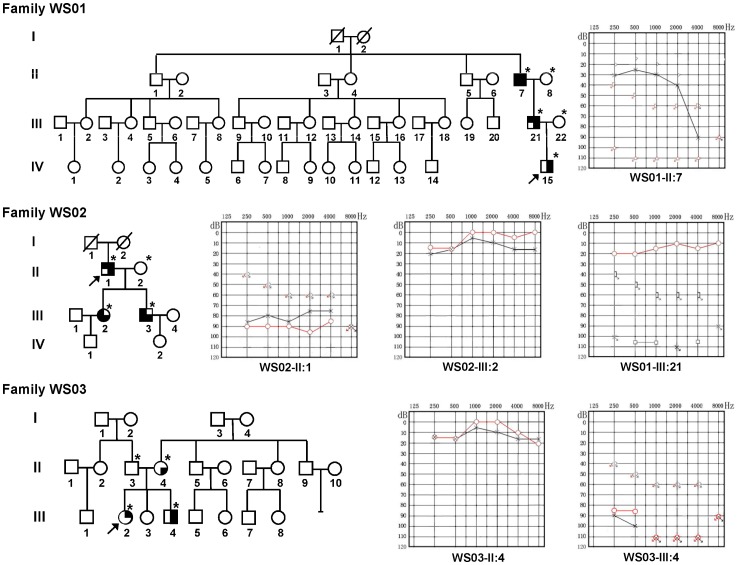
Pedigrees of the Chinese WS2 families WS01, WS02, and WS03, and audiograms of some affected male and female subjects. High clinical variability was observed even within the same family. Not all affected persons manifested all clinical features. Circle, female; square, male; filled quadrants indicate phenotype associated with WS, upper left, premature graying hair; lower left, freckles on the skin; upper right, hearing loss; lower right, heterochromia iridis; arrow, the proband; *, DNA samples available.

In total, 20 WS2 subjects from 14 unrelated families were assessed. The patients consisted of 13 males and seven females, ranging in age from 2–69 years. Among the 14 families, only families WS01, WS02, and WS03 had more than one patient (Fig. 1); the remaining 11 were sporadic cases. Blood samples were obtained from the 20 WS patients, four married-in-control family members, and the parents of the 11 sporadic cases. Additionally, blood samples were obtained from 200 region- and ethnicity-matched volunteers with normal hearing. DNA was extracted from peripheral blood leukocytes using a DNA extraction kit (Watson Biotechnologies Inc., Shanghai, China).

### Clinical evaluation

Twenty WS2 patients were diagnosed according to the criteria proposed by the WS consortium [10]. A comprehensive clinical history and neurotological, ophthalmological, and dermatological examinations were performed on all subjects. The audiological and neurotological examination consisted of otoscopy, pure-tone audiometry, immittance testing, and auditory brain-stem response (ABR). Additional auditory steady-state response (ASSR) tests were performed for young individuals who did not respond well to the pure-tone audiometry test. The ophthalmological examination included visual acuity measurements, visual field examination, and fundus ophthalmoscopy. Special attention was given to the color of skin, hair, and iris as well as developmental defects such as dystopia canthorum and limb abnormalities. The degree of hearing loss was defined according to the pure-tone average (PTA), which was based on three frequencies (500, 1000, and 2000 Hz) as follows: normal, <26 dB HL; mild, 26–40 dB HL; moderate, 41–70 dB HL; severe, 71–90 dB HL; and profound, >90 dB HL.

### Mutational analysis

All coding exons and 200 bp of the flanking intron regions of the WS2-related genes *MITF-M* isoform, *SOX10*, and *SNAI2* were amplified in all patients by polymerase chain reaction (PCR) using specific primers (for details, see the File S1). All PCR amplifications were carried out using 40 ng of genomic DNA and 2 pmol of each primer. PCR conditions were 94°C for 4 min, then 30 cycles of denaturation at 94°C for 30 s, annealing at various temperature for 30 s for the different primers, and extension at 72°C for 30 s, followed by a 7-min final extension at 72°C. PCR fragments were ethanol-purified and sequenced in both directions using the ABI BigDye Terminator Cycle Sequencing Kit (ver. 3.1; ABI Applied Biosystems, Foster City, CA), with the same primers used for PCR. The raw sequence data produced by the ABI Prism 3100 DNA sequencer were aligned with the wild-type sequence using the GeneTool program.

### Bioinformatics Analysis

Phylogenetic conservation of missense mutations was analyzed by aligning the amino acid sequences from several species (retrieved from the Entrez protein database at NCBI) using the ClustalX 2.012 program [11]. *In silico* predictions of the putative functional effects of the missense mutations were conducted with the PMut (http://mmb2.pcb.ub.es:8080/PMut/) [12], Polyphen2 [13] (http://tiddlyspace.com/bags/icgc_public/tiddlers/PolyPhen2), and SIFT (http://sift. jcvi.org) software [14].

## Results

### Clinical findings

All 20 patients were diagnosed with WS2 based on their calculated W index (<1.85) and the absence of musculoskeletal anomalies and intestinal aganglionosis. Among the 20 WS2 cases, deafness and heterochromia iridum were the most frequent features, and 17 patients had sensorineural hearing impairment (17/20, 85.0%), which varied from moderate to profound. The age of deafness onset varied from congenital to 40 years. Additionally, younger patients frequently had congenital, profound, bilateral hearing loss (14/20, 70.0%).

**Figure 2:**
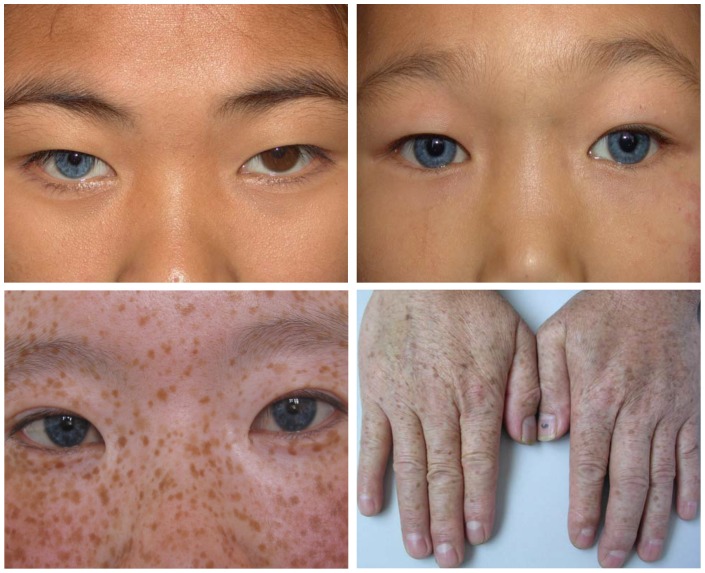
Photographs of some affected individuals. Upper left, the proband of family WS13 presented with complete heterochromia iridis. Lower left, the proband of family WS04 presented with bilateral blue irides and special brown freckles on the face. Upper right, the proband of family WS08 presented with bilateral characteristic brilliant blue irides. Lower right, individual II:7 of family WS01 had numerous freckles on the dorsum of the hands. (The subjects in the photographs provided written informed consent for publication of their photograph, as outlined in PLoS ONE consent form.).

All 20 affected individuals (20/20, 100.0%) had heterochromia iridum, including different-colored eyes in four cases and partial or segmental heterochromia of one or both eyes in eight cases. The characteristic brilliant blue iris, which is more often seen in WS1 cases, was observed in eight of the 20 WS2 patients. A white forelock was observed in two patients (2/20, 10.0%). Premature graying was not assessed due to the young age of most patients. Five affected individuals (5/20, 25.0%) had numerous brown freckles on the face, trunk, and limb extremities (Fig. 2). No patchy or generalized skin depigmentation was observed in any of the patients. Table 1 lists the clinical data of these 20 Chinese WS2 patients.

**Table 1:** Summary of Clinical Data for 20 Chinese WS2 Patients.

*Pedigree*	*Gender*	*Age (years)*		*Iris*	*Skin*	*W*	*Severity of HL*	
		*At Testing At Onset*					*Left ear*	*Right ear*
WS01								
II-7	Male	58	10	A	+	1.80	moderate	profound
III-21	Male	30	10	B	+	1.76	profound	normal
IV-15	Male	3	Prelingual	C	−	1.45	profound	profound
WS02								
II-1	Male	69	40	B	−	1.84	severe	severe
III-2	Female	39	−	B	+	1.82	normal	normal
III-3	Male	36	−	B	+	1.76	normal	normal
WS03								
II-4	Female	36	−	B	−	1.70	normal	normal
III-2	Female	12	Prelingual	B	−	1.65	profound	profound
III-4	Male	6	Prelingual	B	−	1.63	profound	profound
WS04	Female	16	Prelingual	C	+	1.64	profound	profound
WS05	Male	18	Prelingual	C	−	1.76	profound	profound
WS06	Male	14	Prelingual	C	−	1.80	profound	profound
WS07	Male	15	Prelingual	C	−	1.74	profound	profound
WS08	Male	4	Prelingual	C	−	1.43	profound	profound
WS09	Female	9	Prelingual	B	−	1.41	profound	profound
WS10	Male	2	Prelingual	A	−	1.40	profound	profound
WS11	Male	6	Prelingual	C	−	1.75	profound	profound
WS12	Female	7	Prelingual	C	−	1.53	profound	profound
WS13	Female	14	Prelingual	A	−	1.86	profound	profound
WS14	Male	3	Prelingual	A	−	1.60	profound	profound

A, complete heterochromia iridis; B, partial or segmental heterochromia iridis; C, brilliant blue iris; Skin, numerous brown freckles on the face, trunk, and limb extremities; W, W index; HL, hearing loss; +, sign present; −, sign absent.

### Identification of mutations

A heterozygous nonsense mutation, c.763C>T, in *MITF* exon 8 was identified in three affected members (II∶7, III∶21, and IV∶15) of family WS01, resulting in a premature termination codon at 255 within the helix-loop-helix leucine zipper domain of the MITF protein (p. Arg255X). Unaffected married-in-control family members (II∶8 and III∶22) did not have the same mutation.

Three heterozygous missense mutations in *MITF* were identified in the WS2 patients. Heterozygous c.20A>G was identified in subjects II:1, III:2, and III:3 of family WS02 and resulted in the replacement of tyrosine by cysteine at codon 7 (p. Tyr7Cys). Married-in-control family member II:2 did not carry this mutation. Heterozygous c.649A>G (p. Arg217Gly) was found in family WS08. A nucleotide substitution, c.332C>T (p. Ala111Val), was detected in family WS09; this is a known polymorphism in dbSNP (rs182533927). Conservation analyses revealed that the Arg residues at 217 and 255 in MITF are conserved across human, mouse, chicken, cow, and dog.

**Figure 3:**
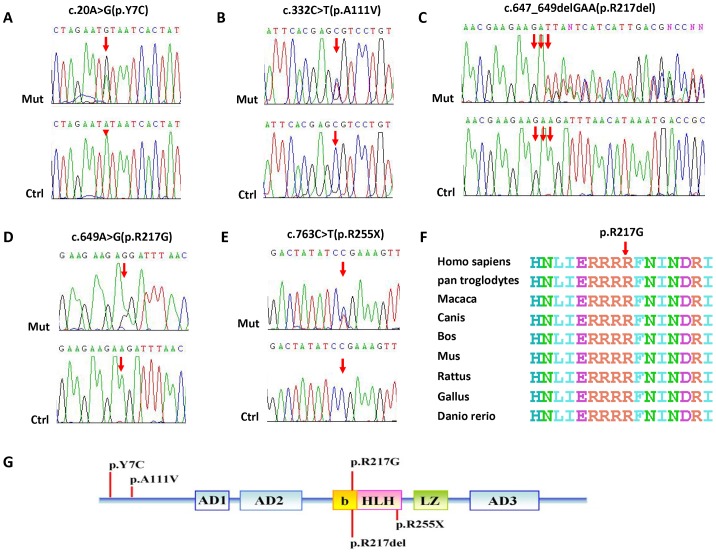
Mutation analyses of Chinese WS2 families WS01, WS02, WS04, WS08, and WS09. A. DNA sequence chromatograms showing heterozygous missense c.20A>G mutation identified in family WS02, compared with wild-type controls. The structure of MITF indicates the position of c.20A>G mutation in exon 1 and p. Y7C outside the HLH domain. B. DNA sequence chromatograms showing heterozygous missense c.332C>T mutation identified in family WS09, compared with wild-type controls. The structure of MITF indicates the position of c.332C>T mutation in exon 3 and p. A111V outside the HLH domain. C. DNA sequence chromatograms showing heterozygous c.647_649delGAA deletion mutation identified in family WS04, compared with wild-type controls. The structure of MITF indicates the position of c.647_649delGAA mutation in exon 7 and p. R217del in the HLH domain. D. DNA sequence chromatograms showing heterozygous missense c.649A>G mutation identified in family WS08, compared with wild-type controls. The structure of MITF indicates the position of c.649A>G mutation in exon 7 and p. R217G in the HLH domain. E. DNA sequence chromatograms showing heterozygous nonsense c.763C>T mutation identified in family WS01, compared with wild-type controls. The structure of MITF indicates the position of c.763C>T mutation in exon 8 and p. R255X in the HLH domain. F. Conservation analysis shows that the Arg residue at 217 in MITF is conserved across human, Pan troglodytes, macaca, canis, bos, Mus musculus, Rattus norvegicus, gallus, and Danio rerio. G. Schematic illustration of *MITF* gene structure showing the position of the mutations. AD1-3, transactivation domains; b, basic domain; HLH, helix-loop-helix domain; LZ, leucine zipper domain.

A heterozygous c.647_649delGAA deletion was identified in family WS04 and caused the deletion of one of a run of four arginines in the basic domain of the MITF protein. Although the DNA and protein sequences did not reveal which of the four arginines was deleted, we named this mutation p. Arg217del, based on the literature. The unaffected parents of family WS04 did not carry this mutation. None of these five *MITF* mutations was found in the 200 unrelated normal controls. Figure 3 presents the chromatograms of five mutations. We did not detect any mutations in the *SOX10* or *SNAI2* genes.

### *In silico* analysis of missense substitutions

The p. Tyr7Cys and p. Ala111Val mutations fell outside of the important MITF domains, and were predicted to have a neutral effect by the PolyPhen-2, PMut, and SIFT software, which indicated ‘benign’, ‘neutral’, and ‘tolerated’, respectively. The missense substitution p. Arg217Gly fell within the MITF helix-loop-helix domain, and was predicted to have a pathogenic effect by the PolyPhen-2, PMut, and SIFT software, which indicated ‘probably damaging’, ‘pathological’, and ‘affect protein function’, respectively. We also examined the evolutionary conservation of the mutated residues and surrounding amino acids. This analysis revealed that arginine at position 217 in MITF is highly conserved in different species (Fig. 3).

## Discussion

Sensorineural hearing loss is the most common feature of WS2, and is also the main reason motivating patients to visit a physician. Hageman and Delleman, who reviewed 159 cases in the literature, reported that deafness occurred in 57% of WS2 cases [15], and Liu *et al.* reported a rate of 77% (62/81) [3]. We found a similarly high rate of 85.0% (17/20) in these Chinese WS2 patients, and found that the severity of deafness varied within and between families, ranging from moderate to profound. Bilateral hearing loss (16/20, 80.0%) was more frequent than unilateral hearing loss (1/20, 5.0%). Age at onset varied from congenital to postlingual hearing loss, with congenital, bilateral profound hearing loss frequently observed in teenagers. Heterochromia iridum was observed in 100.0% (20/20) of the patients in our study, much higher than the rates reported by Liu *et al.* in a British population (40/81, 49.4%) [3] or by Hageman and Delleman (73/159, 46.0%) [15]. Heterochromia iridum included partial or segmental heterochromia of one or both eyes in eight cases (8/20, 40.0%), eyes of different color in four cases (4/20, 20.0%), and the characteristic brilliant blue iris in eight cases (8/20, 40.0%). Our results support the findings of Liu *et al.* [3]: the characteristic brilliant blue iris often seen in WS1 is not a rare clinical manifestation of heterochromia iridum in WS2. Skin pigmentary abnormalities were less frequent (5/20, 25.0%), and generally appeared as numerous brown freckles on the face, trunk, and limb extremities. Chen *et al.* reported 13 Chinese WS2 cases, five of whom manifested very similar skin pigmentary abnormalities [16]. Instead of patchy, depigmented skin, as seen in most Western cases, numerous brown freckles on the skin might be a more common phenotype of skin pigmentary abnormalities in Chinese WS2 patients.

MITF (microphthalmia-associated transcription factor) protein is a member of the helix-loop-helix leucine zipper (b-HLH-Zip) transcription factor family and is the key transcription factor for melanocyte development [17]. Through binding specific DNA sequences, MITF also regulates the transcription of several key melanocytic genes, including tyrosinase (TYR), tyrosinase-related protein 1 (TRP1), and TRP2 (also known as DCT) [18], [19]. To date, fewer than 80 *MITF* and *mitf* mutations have been identified in human and mouse alleles (MITF homepage-LOVD-Leiden Open Variation Database, <http://grenada.lumc.nl/LOVD2/WS/>; Mouse Genome Database, <http://www.informatics.jax.org>). Among these, fewer than 40 *MITF* mutations have been identified in a number of human WS2 and Tietz syndrome families, and most were private mutations, except for c.33+1G>A, c.640C>T (p. Arg214X), c.775C>T (p. Arg259X), and c.649_651delAGA (p. Arg217del). Several human mutations are identical to mouse *mitf* alleles, including p. Arg217del (*mi* allele in mouse), p. Arg216Lys (*mi^or^* allele in mouse), and p. Ile224Ser (*mi^enu122^* allele in mouse). The majority of these mutations are located in exons 7 and 8 of the *MITF* gene; these encode the b-HLH-Zip domain. The b-HLH-Zip domain makes sequence-specific DNA contacts with the basic region of the domain and mediates the homo- and heterodimeric interactions necessary for DNA binding. Interruption of the b-HLH-Zip domain binding decreases the ability of the mutant MITF protein to bind to the CATGTG core DNA sequence in the human tyrosinase promoter [20].

In this study, we identified five *MITF* variations: p. Tyr7Cys, p. Ala111Val, p. Arg217Gly, p. Arg217del, and p. Arg255X. Missense mutations, p. Tyr7Cys and p. Ala111Val, are more likely have neutral effects, while p. Arg217Gly, p. Arg217del and p. Arg255X appear to be more likely to have disease-causing effects. The total mutational frequency of the *MITF* gene in our study was 21.4% (3/14), higher than the rate of 15% reported among patients of Western descent [2]. The higher *MITF* mutational frequency in our study may be partly due to bias associated with the small number of samples we tested. A larger cohort of WS2 patients should be recruited for future studies. In the literature, we found three *de novo MITF* gene mutations in 14 Chinese WS2 cases for which only exons and flanking splicing sites had been sequenced [16], [21]. Thus, we propose creating a *MITF* mutation database for the Chinese population. Table 2 summarizes the *MITF* gene mutations identified in Chinese WS2 patients.

**Table 2:** Summary of *MITF* Gene Mutations Identified in Chinese WS2 Patients.

*No.*	*Nucleotide change^a^*	*Amino acid change^b^*	*Exon*	*References*
1	c.20A>G*	p. Tyr7Cys*	1	This study
2	c.332C>T*	p. Ala111Val*	3	This study
3	c.575delC*	P. Thr192LysfsX20*	6	[16]
4	c.639delA*	p. Glu213AspfsX8*	7	[21]
5	c.647_649 del GAA	p. Arg217del	7	This study
6	c.648_650delAAG	p. Arg217del	7	[16]
7	c.649A>G*	p. Arg217Gly*	7	This study
8	c.650G>T*	p. Arg217Asn*	7	[16]
9	c.763C>T*	p. Arg255X*	8	This study

a. Description of the mutations is based on the GenBank reference sequence for the *M* isoform of the *MITF* gene: NM_000248.3.

b. Amino acid numbering is based on GenBank Reference Sequence: NP_000239.1.

* , mutations first described in a Chinese population.

The English in this document has been checked by at least two professional editors, both native speakers of English. For a certificate, please see: http://www.textcheck.com/certificate/O0uNHd.

The p.Arg255X mutation appears to result in a truncated MITF protein, lacking the 3′-end of the helix-loop-helix domain, the whole leucine zipper domain, and the third transactivation domain. Mutant MITF proteins are thought to have defects in homo- or heterodimerization and DNA binding through their basic regions, as well as DNA binding with related transfer factors (TF), such as TFE3, TFEB, and TFEC [22].

The missense substitutions of p. Tyr7Cys and p. Ala111Val occurred outside the functional domain of the MITF protein. The exact pathogenic mechanisms of these two mutations remain unclear without functional tests in *vivo*, but the bioinformatics analyses indicated that they may have neutral effects.

The recurrent mutation p. Arg217del has been reported in several WS2, WS2 with ocular albinism (OA), and Tietz syndrome families [9], [16], [23]–[25]. The mutation in Tietz syndrome and WS2 with ocular albinism (OA) was c.649_651delAGA, while in Chinese WS2 patients the mutations were c.648_650delAAG and c.647_649delGAA, as reported by Chen et al. [16] and based on our results. From the DNA and protein sequences, it was difficult to determine which of a run of four arginines was deleted; however, deletion of one of the four arginines occurred within the basic domain, a critical region that plays a significant role during the homo- or heterodimerization of MITF binding to DNA. Its recurrence might be partly due to the presence of a short nucleotide triplet repeat. Functional tests revealed that the *mi* allele, identical to the human p. Arg217del mutation, impaired nuclear localization of the resulting protein and had an inhibitory effect on the nuclear localization potential of wild-type MITF [26]. The mutation p. Arg217Gly also occurred in the basic domain of MITF and appears to decrease the DNA binding ability of MITF and have phenotypic consequences. The p. Arg216Lys mutation in humans is identical to the mouse *mi* ^or^ allele, which abrogates DNA binding to E-boxes and precludes binding of wild-type MITF homodimers [22]. In the cohort investigated by Chen *et al.*, two mutations, p. Arg217Ile and p. Arg217del, occurred at the same nucleotide position, 217, in the MITF protein, and four families were affected (4/13) [16]. In our study, two mutations, p. Arg217Gly and p. Arg217del, were identified in two affected families (2/14). Thus, we speculate that the total mutational frequency of the *MITF* gene at codon 217 is 22.2% (6/27) among Chinese WS2 patients, and so the first step of *MITF* mutation screening should include codon 217.

## Conclusions

In this study, we conducted clinical evaluations and mutation identification of the *MITF* gene in 20 Chinese WS2 patients from 14 families. The molecular basis of 78.6% of WS2 cases remains unclear, and no correlation appeared between WS2 phenotype and genotype. Based on the high rates of *MITF* mutation in our Chinese WS2 patients, a *MITF* mutation database for the Chinese population should be created. Future research should examine more WS2 cases, employ new techniques to identify new mutations in the known genes and identify other possibly related genes, and focus on functional studies using animal models.

## Supporting Information

File S1 (DOC)
