# Supplementary material for: Genetic and Phenotypic Heterogeneity in Chinese Patients with Waardenburg Syndrome Type II
Source: PLoS One. 2013 Oct 23;8(10):e77149. doi: 10.1371/journal.pone.0077149 (PMC3806753; doi:10.1371/journal.pone.0077149)
Supplement: File S1 — (DOC) [file pone.0077149.s001.doc]

| **Table S1. PCR primers for amplification of *MITF-M* isoform exons** | | | |
| --- | --- | --- | --- |
|  | | | |
| *Exon* | *Forward primers*  *(5’→3’)* | *Reverse primers*  *(3’→5’)* | *PCR fragment*  *(bp)* |
| 1 | TGGTGTCTCGGGATACCTTG | TGGCATCAAATAATAAACAGCA | 304 |
| 2 | TGGCATCAAATAATAAACAGCA | GTGGCCACAAGGACAAACTA | 429 |
| 3 | CATCTTGTTGCTGTGCCATC | AAGGTGTGATCCACCACAAA | 253 |
| 4 | GACCATTATTGCTTTGGGTAAAA | TGTGATCCTGAGATAATTCTCCATT | 343 |
| 5 | TGAGGAGATCCTGTACCTCTCTT | AAAAGTTACGTCCATGAGTTGGA | 425 |
| 6 | GCTTTTGAAAACATGCAAGC | GCTGTAGGAATCAACTCTCCTCT | 350 |
| 7 | CGTTGTCATGACCTGGAGAA | CAAAGGGAGAGGGGAGACTT | 301 |
| 8 | CTTATCCATGTAACCAAGCA | CACACACACAGAATCCACAAA | 425 |
| 9 | CTAATGACGCGCATCTACCA | TCCTGGGCTATTGATAAAGCA | 646 |
|  | | | |

| **Table S2. PCR primers for amplification of *SOX10* exons** | | | |
| --- | --- | --- | --- |
|  | | | |
| Exon | Forward primers  (5’→3’) | Reverse primers  (3’→5’) | PCR fragment  (bp) |
| 3 | GTGGGCGTTGGACTCTTTGC | GCCTCGGCTACCCTGAATCC | 578 |
| 4 | CCCAGGGCCTCACATCTTCC | CATTGCCATCCAGCCATCTCC | 453 |
| 5a | ACCTGCCTCTAACCTGCTTCC | ATAATAGGGTCCTGAGGGCTGATG | 597 |
| 5b | ACTACACCGACCAGCCATCC | GCAGTGAGCCAGACAGAAAGC | 452 |
|  | | | |

| **Table S3. PCR primers for amplification of *SNAI2*exons** | | | |
| --- | --- | --- | --- |
|  | | | |
| Exon | Forward primers  (5’→3’) | Reverse primers  (3’→5’) | PCR fragment  (bp) |
| 1 | GCTGTGATTGGATCTTTCTTGC | TGTAAGCTCCCTTTCAGGACAC | 450 |
| 2 | TGTGTGTATACTTGCGTGTGG | CTTCATGCAAATCCAACAGC | 700 |
| 3 | ATTTCTGTATGATTGGCAGCAG | AGCTTCGGAGTGAAGAAATGC | 470 |
|  | | | |
